# Examination of medical student and physician attitudes towards suicide reveals need for required training

**DOI:** 10.3389/fpubh.2024.1331208

**Published:** 2024-04-03

**Authors:** Paulyna Schulz, Isain Zapata, Teodor Huzij

**Affiliations:** ^1^Rocky Vista University College of Osteopathic Medicine, Englewood, CO, United States; ^2^Department of Biomedical Sciences, Rocky Vista University College of Osteopathic Medicine, Englewood, CO, United States; ^3^Department of Osteopathic Principles and Practice, Rocky Vista University College of Osteopathic Medicine, Englewood, CO, United States

**Keywords:** suicide prevention, medical education, suicide attitudes, medical student, residency education, awareness, physician

## Abstract

The attitudes of healthcare providers towards suicidal patients are known to influence their motivation to treat patients during a suicidal crisis. Patients who attempted suicide are more likely to have recently visited a primary care provider who is not necessarily sufficiently trained in managing a suicidal patient rather than a mental health provider who is trained to do so. For those reasons, documenting medical students and physicians’ attitudes towards suicide can help in the development of effective intervention training to prepare them to manage these types of patients. In this mini review, attitudes towards suicidal patients, the effectiveness of training on changing their attitudes are discussed. In summary, primary care providers are recognized as a top area where improvements can prevent suicides; providing proper suicide prevention training can effectively improve attitudes and quality of care for suicidal patients.

## Introduction

The seriousness and magnitude of the suicide epidemic continues to worsen as it holds the 12th leading cause of death in the United States and the 17th leading cause of death worldwide (1, 2). More seriously, suicide remains the second leading cause of death for ages 10–14 and 25–34 (1). In 2020, approximately 46,000 people in the U.S. died by suicide; this means every 11 min, a person dies by suicide (1).

Patients who attempt suicide are more likely to have visited their primary care provider than a mental health provider within 1 month of their suicide attempt (3, 4). Eighty percent of youth who die by suicide saw their primary care provider within 1 year of their death (5). Physicians serve as potential gatekeepers of suicide prevention as they are more likely to have contact with high-risk suicidal patients, however, most are not formally trained to assess and recognize the risks of suicidality (6, 7). Care for suicidal patients relies heavily on a physician’s ability to judge a patient’s suicide risk while implementing effective communication with empathy and compassion and providing a safe place with nonjudgmental listening (6). An international study reveals that these skills are not adequately being utilized in practice (8, 9). Patients who attempted suicide were asked about their experience of care with healthcare providers and the responses were predominantly negative despite differences in culture and healthcare systems. Patients reported experiencing inappropriate staff behavior, poor communication, decisions being made without patient involvement, feeling the staff lacked knowledge to care for them and lack of direction for care after discharge (8, 9). Such experiences reveal the lack of quality of care that suicidal patients may be receiving when trying to seek help from physicians.

Literature reveals that attitudes of healthcare providers towards suicidal patients influences the motivation or lack thereof to treat patients during a suicidal crisis (10, 11). The objective of this literature review is to document attitudes medical students and physicians have towards suicide, how these attitudes influence care for suicidal patients and how suicide intervention training can be effective in changing attitudes and quality of care for suicidal patients. This study was not designed as a scoping or systematic revision of literature. The significance in studying this topic is to provide an understanding of how future and current physicians respond to suicidality and how it influences their care for suicidal patients. It also addresses the lack of formal training medical students and physicians receive about suicide intervention and how this lack of training may play a significant role in influencing the attitudes presented and the lack of care patients receive. This relationship between attitudes and approach to care is significant because negative attitudes towards patients with mental illness and suicidality leads to detrimental outcomes like delaying care, not providing appropriate care to patients with help seeking behavior, and not assessing for suicidality in a patient presenting with suicidal risk factors (11).

### Medical students and physicians’ attitudes towards suicidal patients

Currently, there is little to no required training for medical students about suicidality in the medical school curriculum (5, 12). For most medical students, their exposure to suicidal patients and their care is limited to their required one-month psychiatry clerkship. This limited exposure has no effect in changing the attitudes that medical students have towards suicide as their attitudes remain the same pre-and post-clerkship (11).

Few studies have been conducted examining medical students’ attitudes towards suicidal patients (10, 13–16). Cultural differences have been noted when comparing medical students internationally, however, predominantly negative attitudes were found regardless of location and nationality of the student (10, 13–16). Most medical students’ attitudes are consistent with beliefs that suicide attempters are lonely and depressed, and that suicidal behavior is related to weak personality and self-destructive behaviors (10). Attitudes among students seems to be influenced by knowledge of mental disorders and personal experiences (16). An association has been found between students’ firsthand experiences with suicide or previous contact with individual(s) with mental illness and an increase in comfort and confidence in caring for individuals with suicidal behaviors (15, 17). Final year students report an increased understanding that patients who have attempted suicide are at higher risk of another attempt and that patients who do complete suicide are not responsible for their actions (16). The same students also demonstrated a strong belief that suicidal patients can be helped, and that suicide can be prevented (16). Despite these reports, most surveyed students are very uncertain about their attitudes towards suicide which may imply lack of awareness and education (10).

Physicians’ attitudes towards suicide are more positive when compared to students. This may be influenced by the increased level of knowledge and awareness towards patient autonomy (9, 11, 18). Psychiatrists are found to have the highest level of positive attitudes and greater willingness to help compared to other physicians (9, 11). Research further reveals medical providers have similar attitudes toward suicide as non-medical personnel. This suggests physicians consider suicidality similarly to the general public (18). This similarity may explain why attitudes prevent physicians from initiating intervention when encountering a suicidal patient, especially when attitudes have been found to be influenced by the level of education about suicide. This suggests a physician’s training in suicidality is equal to that of the public, which is little to none.

Personal experience with suicidal thoughts or loss of a loved is correlated with an increased understanding and empathy for suicidal patients (11). Such experiences can improve a physician’s ability to recognize risk factors for suicide and their knowledge of mental illness and suicidal ideations (11). Physicians who had a personal experience with depression and/or suicidal behaviors are less likely to present with negative and stigmatizing attitudes and are more likely to be aware of suicidal behavior among their patients (11). Physicians with previous experience working with suicidal patients are more likely to address the topic of suicide with a patient. However, it has been shown that physicians who have a high number of patient losses to suicide are more likely to experience feelings of incompetency and doubt in their ability to prevent suicide (11).

The data gathered about attitudes of medical students and physicians towards suicide reveals most have a lack of awareness and understanding of suicidality, especially among students (10, 16). Majority of students reported predominantly negative attitudes which can lead to lack of care and suicide prevention (11, 14). The negative attitudes reported by physicians appear to be related to decreased confidence levels in addressing suicidality with patients (9, 11, 19). Negative attitudes and lack of confidence may be due to lack of experience of working with suicidal patients, but data suggests it is more likely due to a lack of intervention training (11, 18, 20–23).

### Effectiveness of training on changing attitudes and quality of care

The effectiveness of training among medical students and primary care providers was investigated because data collected suggests that lack of formal training and education may serve as the primary factor influencing attitudes and lack of quality of care for patients. Surveyed medical students agree that they have a responsibility in preventing suicide, however, half of the students admitted they were not comfortable working with suicidal patients (20, 21). A three-hour education intervention for the same medical students was found to improve attitudes toward suicide prevention especially in their ability to assess suicidal ideations and identifying attributes of suicidality. Prior to the intervention, most students believed that suicide was an attention seeking behavior; this belief changed to suicide being a cry for help after the intervention. Post-training, students reported feeling less resentment and more responsibility and competence in working with suicidal patients. The post-intervention results revealed that limited focus on suicide during medical school and the deficit in skills and confidence in addressing suicide may contribute to the negative beliefs about suicide (21).

Increasing knowledge through training and improving the perception of being sufficiently trained consistently shows improvement in positive attitudes towards suicide among healthcare providers (11, 22). Trainings also help increase knowledge and self-confidence which leads to a greater likelihood of screening for suicidal ideations and performing risk assessments (11, 23). Physicians who have participated in trainings report feeling less afraid to ask about suicide, have an increased ability to recognize suicidal risk factors and behaviors, and have a greater understanding of the correlation between mental illness and suicidality (18, 23).

Physicians demonstrate accuracy in diagnosing depression severity, however, only one-third report they would confidently conduct proper suicide risk assessment for their depressed patients (6, 24). The hesitancy to conduct a suicide risk assessment is not due to lack of knowledge of suicide risk factors but is due to decreased confidence in accurately identifying these factors in patients (25). Implementation of proper suicide prevention training for medical students and physicians is necessary because assessing for suicide risk is not quantifiable and requires an intentional clinical interview to appropriately gauge suicide risk (26). Preventing suicide relies on a physician’s ability to judge suicidal risk accurately with foundational knowledge and skills (6). Surveyed primary care providers report current medical and residency training do not adequately prepare them to identify imminently suicidal patients (7). Residency program directors and their residents report similar opinions and endorse the need for standardized curriculum in suicide prevention training as what is currently available is not sufficient (7). Integrating early suicide prevention training during medical school significantly improves students’ abilities to implement suicide intervention strategies and increases their confidence in discussing suicidality with patients (27). The implementation of early and continued training has the potential to positively impact the outcome of suicidal patient management in primary care (27).

## Discussion

Literature reveals medical students present with predominantly negative attitudes toward suicide and lack confidence in addressing suicidal patients. Despite progression through medical and residency training, physicians report similar beliefs and negative attitudes unless they have personal experience related to suicide or practice psychiatry. The negative attitudes reported by physicians appear to be primarily related to decreased confidence levels in addressing suicidality with patients. The presence of such negative attitudes plays an impactful role in preventing primary care providers from appropriately conducting suicide risk assessment with their patients. This barrier is negatively impacting quality of care for high-risk patients, especially when considering these patients are more likely to seek help from their primary care provider than a mental health provider within 1 month of their suicide death.

The overall impact of specific laws and legislation for suicide prevention have been evaluated in a large systematic review that highlights the benefits of restricting access to lethal means, school suicide awareness preventions programs and careful assessment of pharmacological risk but highlight the lack of healthcare provider prevention approaches (17). In the United States there is no medical education specific legislation on suicide prevention training. Screening during healthcare encounters is not done routinely (28). Suicide prevention in medical education is based on Center for Disease Control and Prevention (CDC) and National Institute of Health (NIH) or American Foundation of Suicide Prevention (AFSP) recommendations. Legislation has been passed in some states requiring or encouraging suicide prevention in schools (17, 29).

Unfortunately, the efforts of increasing awareness about suicide appear not to be sufficient to influence physicians to actively prevent suicide among their patients. This is evident by the reality that physicians’ attitudes are like the general public where increased awareness about suicide is the primary strategy to increase education and intervention for suicide. The AFSP identified that primary care serves as one of three settings where missed opportunities to prevent suicide can be effectively replaced to become evidence-based risk-reducing interventions that can save lives (12). Further research reveals that formal medical student and physician training in suicide prevention is among the top three most promising strategies to reduce suicide rates (30). Although physicians are viewed as gatekeepers in preventing suicide, many of them hold negative attitudes towards suicide and are not formally trained to accurately assess and recognize risk factors in their patients (6). Literature overwhelmingly shows that medical students and physicians with proper suicide intervention training can present with more positive attitudes towards suicide and provide better quality of care for suicidal patients that can play a significant role in preventing suicide and ultimately save lives.

## Author contributions

PS: Conceptualization, Investigation, Writing – original draft, Writing – review & editing. IZ: Conceptualization, Supervision, Writing – review & editing. TH: Conceptualization, Supervision, Writing – review & editing.
